# 3D Printing of Cytocompatible Graphene/Alginate Scaffolds for Mimetic Tissue Constructs

**DOI:** 10.3389/fbioe.2020.00824

**Published:** 2020-07-17

**Authors:** Jianfeng Li, Xiao Liu, Jeremy M. Crook, Gordon G. Wallace

**Affiliations:** ^1^ARC Centre of Excellence for Electromaterials Science, Intelligent Polymer Research Institute, AIIM Facility, University of Wollongong, Wollongong, NSW, Australia; ^2^Illawarra Health and Medical Research Institute, University of Wollongong, Wollongong, NSW, Australia; ^3^Department of Surgery, St Vincent’s Hospital, The University of Melbourne, Fitzroy, VIC, Australia

**Keywords:** 3D bioprinting, graphene, alginate, adipose stem cell, bone, biomaterials, regenerative medicine, instructive scaffolds

## Abstract

Tissue engineering, based on a combination of 3D printing, biomaterials blending and stem cell technology, offers the potential to establish customized, transplantable autologous implants using a patient‘s own cells. Graphene, as a two-dimensional (2D) version of carbon, has shown great potential for tissue engineering. Here, we describe a novel combination of graphene with 3D printed alginate (Alg)-based scaffolds for human adipose stem cell (ADSC) support and osteogenic induction. Alg printing was enabled through addition of gelatin (Gel) that was removed after printing, and the 3D structure was then coated with graphene oxide (GO). GO was chemically reduced with a biocompatible reductant (ascorbic acid) to provide electrical conductivity and cell affinity sites. The reduced 3D graphene oxide (RGO)/Alg scaffold has good cytocompatibility and can support human ADSC proliferation and osteogenic differentiation. Our finding supports the potential for the printed scaffold’s use for *in vitro* engineering of bone and other tissues using ADSCs and potentially other human stem cells, as well as *in vivo* regenerative medicine.

## Introduction

Tissue engineering involves reconstruction and/or functional recovery of malfunctioned tissue (Amini et al., 2012; Dai et al., 2016; Han and Du, 2020). 3D biocompatible scaffolds serve to provide cell support by facilitating native extracellular matrix formation, promoting cell growth, and if necessary, differentiation (Kim et al., 2015; Li et al., 2019a). More specifically, optimally porous scaffolds provide channels for diffusion of exogenously delivered and endogenous cell-secreted bioactive factors, mechanical support for maintaining tissue dimensions, and an ECM-like environment ahead of native ECM production (Hollister, 2005). Human ADSCs presently employed have significant potential for autologous transplantation in tissue engineering, being easily accessible, self-renewable and able to differentiate into multi-lineage cell types, such as bone, skeletal, muscle, adipose, and cartilage cells (Bunnell et al., 2008).

Alginate (Alg), a polysaccharide extracted from brown algae, has been applied in various bio-related fields due to its cytocompatibility and attractive physicochemical properties (Yang et al., 2011; Lee and Mooney, 2012). Efficient gelation of Alg by simple addition of divalent cations makes it an ideal candidate for 3D bioprinting (Song et al., 2011; Valentin et al., 2019). Printed Alg structures have been used to engineer various human tissues such as aortic valve (Hockaday et al., 2012), cardiac tissue (Gaetani et al., 2012), bone tissue (Wang et al., 2015), and blood vessels (Shi et al., 2015; Yeo et al., 2016). However, Alg structures are not mechanically robust, resulting in printing failure or inaccurate geometries (He et al., 2016). In addition, Alg is an inert material with respect to critical cell adhesion and proliferation (Kim et al., 2016). Notwithstanding limitations, Alg can contribute toward a biomimetic environment of 3D printed constructs, with structural control provided by material-combinations such as with gelatin (Gel), enabling useful microscale features through to macroscale architecture, suitable porosity and pore size of scaffolds, all of which are important for tissue formation and regeneration (Nava et al., 2016).

Graphene is being intensely researched globally for applications in fields from electronics to medicine (Geim and Novoselov, 2007; Thompson et al., 2015; Ke and Wang, 2016; Kandyba et al., 2018; Li et al., 2019b). In medicine, the realization of 3D cell-supporting structures containing graphene has significant potential for tissue engineering and replacement. For example, coating graphene onto 3D printed scaffolds should endow 3D structures with mechanical strength and cytocompatibility (Li et al., 2017). In addition, graphene-based structures have been shown to support *in vivo* bone regeneration by providing physicochemical cues and through the enhancement of material biocompatibility (Liu et al., 2019; Palmieri et al., 2020).

Here, we describe the fabrication of 3D biocompatible graphene-coated scaffolds using 3D printing and a bioink comprising Alg and Gel. Following printing, Gel is dissolved and the ensuing Alg scaffold coated with graphene oxide (GO) that is reduced to conductive RGO. The modulus of 3D RGO/Alg scaffolds increased 3.8-fold compared to Alg-only scaffolds. Moreover, 3D RGO/Alg supported ADSC growth and osteogenic induction, with augmented cell proliferation and differentiation toward osteogenic lineage compared with 3D Alg-only scaffolds. Our findings provide proof-of-concept for use of the printed scaffolds for bone engineering and adaptability to a multiplicity of cells and tissues for research and translation, including tissue replacement therapy and regenerative medicine.

## Experimental Section

### Materials

Medium viscosity sodium alginate from brown algae [*M*_W_: 80,000–120,000 Da, ratio of mannuronic acid and guluronic acid (M/G ratio): 61:39; viscosity ≥2,000 cP for 2% w/w solution, 25°C], gelatin (Gel) from bovine skin, L-ascorbic acid-2-phosphate, dexamethasone, β-glycerophosphate and sodium nitrate (NaNO_3_) were purchased from Sigma-Aldrich (United States). Graphite powder was purchased from Aladdin Ltd. (China). Ninety-eight percentage sulfuric acid (H_2_SO_4_), potassium permanganate (KMnO_4_), calcium chloride dihydrate (CaCl_2_⋅2H_2_O), 32% hydrochloric acid (HCl), and 30% hydrogen peroxide (H_2_O_2_) were purchased from Chem-Supply (Australia). L-ascorbic acid was purchased from BDH Chemicals (Australia) and 18 MΩ Milli-Q water was used in all the experiments. RGO structure was prepared by previously reported method (Li et al., 2019c).

### Alg/Gel Ink Preparation

200 mg Alg and 300 mg Gel were dissolved in 9.500 g water with mechanical mixing at 80°C for 3 h, and then the 2%/3% Alg/Gel ink mixture was transferred into a syringe barrel (Nordson EFD, United States) with removal of air bubbles inside by centrifugation (Thermoline K241 centrifuge, Australia). 2% Alg solution was prepared with 200 mg Alg dissolved in 9.800 g water, following the same procedure.

### Rheology

Rheological properties of bioink were tested on an AR-G2 rheometer (TA Instruments, United States) at room temperature (RT; 25°C). Ink was prepared 1 day prior to rheology testing and characterized by using 2°/15 mm steel cone and plate geometry. Both storage modulus (G′) and loss modulus (G′′) were measured as a function of angular frequency during dynamic frequency sweep.

### Synthesis of GO

Modified Hummers method was used to synthesize GO according to previous reported method (Li et al., 2019c). Briefly, 1.0 g graphite was dispersed in 75 ml concentrated sulfuric acid by magnetic stirring in an ice bath, followed by slow addition of 0.5 g sodium nitrate. 2.5 g potassium permanganate was then added over a period of 1 h under vigorous agitation. After 5 days reaction at RT with stirring, 150 ml 5% sulfuric acid solution was added to the reaction system with subsequent heating (90°C for 2 h). 30% hydrogen peroxide was added to remove unreacted manganese dioxide and potassium permanganate after cooling down to RT. The reaction mixture was washed with 1 M hydrochloric acid several times, and then further purified by 1 week dialysis (molecular weight cut-off: 14,000 Da). Obtained graphite oxide was exfoliated to GO by 5 h ultrasonication (Unisonics cleaner, Australia) and 4.5 mg/ml GO aqueous dispersion was prepared afterward. Obtained GO solution was stable for long period of time (more than half year).

### Scanning Electron Microscopy

Morphology of synthesized RGO and 3D RGO/Alg scaffolds was characterized by JEOL JSM-6490LV scanning electron microscope (SEM). GO solution was deposited and dried on glass slides before SEM imaging. For RGO sample imaging, the dried GO samples were chemically reduced by 50 mM L-ascorbic acid solution (80°C, 3 h) and dried. For 3D Alg scaffolds and 3D RGO/Alg scaffolds with or without cells, scaffolds were frozen in liquid nitrogen for 36 s and then characterized by using the JEOL JSM-6490LV SEM directly.

### Raman Spectroscopy

For Raman testing, GO film was obtained by drying GO solution on glass slides that were then subjected to chemical reduction in 50 mM L-ascorbic acid solution at 80°C for 3 h. Obtained GO and RGO films were tested by using Jobin Yvon Horiba HR800 Raman spectrometer (excitation laser λ = 632.8 nm) with 300-lines mm^–1^ grating. 3D RGO/Alg scaffolds were similarly tested.

### Mechanical Testing

Modulus of scaffolds was calculated by compressive testing with wet scaffolds at RT by an EZ-S mechanical tester (Shimadzu, Japan). All the measurements were made with 10 N loading sensor and 1 mm/min compression speed. About 75% strain was applied to the scaffolds during testing. Mean and standard deviation (SD) were calculated from three replicate experiments.

### 3D Printing of Alg/Gel Scaffolds and Coating With Graphene

**FIGURE 1:**
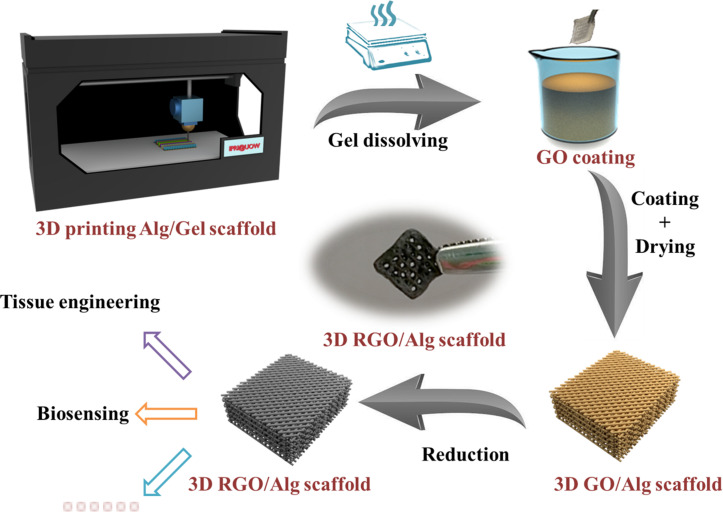
Schematic of 3D RGO/Alg scaffold fabrication.

The process of Alg/Gel scaffold 3D printing and graphene coating is demonstrated in Figure 1. 3D Alg/Gel scaffolds were printed layer-wisely using a 3D Bioplotter machine (EnvisionTEC GmbH, Germany), as previously described (Chung et al., 2013). First, Alg/Gel ink was loaded into a syringe barrel with a 200 μm diameter nozzle (Nordson EFD, United States) fitted and kept at RT for printing. The 3D cubic model (10 mm × 10 mm × 2 mm) comprised 19 layers of Alg/Gel ink, extruded layer-by-layer onto a Petri dish maintained at 5°C. Printing was performed at a feeding speed of 10 mm/s with a strand spacing of 1.5 mm. Extrusion force was generated by air pressure (5 bar) while plotting of 2%/3% Alg/Gel ink.

3D printed Alg/Gel scaffolds were ionically cross-linked with 2% (w/w) CaCl_2_ aqueous solution for 10 min. The Gel component of the scaffold was dissolved by submersion in copious water at 80°C for 3 h.

Obtained 3D Alg scaffolds were wiped with tissue paper (Kimberly-Clark, Australia) to remove surface water and loaded with 10 μl 4.5 mg/ml GO solution. Alg scaffold with GO solution was flipped and squeezed serval times to achieve uniform deposition of GO on the surface. The obtained GO/Alg scaffolds were subsequently dried overnight in air and reduced in 50 mM L-ascorbic acid solution at 80°C for 3 h to produce 3D RGO/Alg scaffolds.

### Electrical Resistance Testing

Assessment of sheet resistance of 3D RGO/Alg scaffolds was conducted with a 4-point probe system (Jandel RM3, United Kingdom). After chemical reduction, 3D RGO/Alg samples were wiped dry with tissue paper and dried in air for 5 h before analysis. A 4-point probe was carefully placed on the RGO coating of the sample and measurements were performed in triplicate.

### ADSC Culture

Human ADSCs (Lonza Corporation, Australia) were cultured in cell growth medium (GRO medium) prepared from 1% 100 × Non-Essential Amino Acids (NEAA) solution, Gibco Dulbecco’s Modified Eagle Medium (DMEM), 1% 100 × penicillin-streptomycin, 10% fetal bovine serum (FBS), 1 ng/ml basic fibroblast growth factor (bFGF) (all from Thermo Fisher, Australia) in a cell culture incubator with a humidified 5% CO_2_ atmosphere environment at 37°C. For cell subculture, an initial cell density of 2 × 10^4^ cells/cm^2^ was used.

### ADSC Seeding on the 3D Scaffold

3D RGO/Alg scaffolds were immersed in GRO medium overnight prior to cell seeding, followed by addition of fresh ADSC GRO medium. Culture medium was refreshed every 2 days.

### Scaffold Cytocompatibility Analysis

Live/Dead assay was made of 5 μg/ml Calcein AM (Thermo Fisher, Australia) and 1 μg/ml propidium iodide (PI; Thermo Fisher, Australia) according to manufacturer’s protocol. After 7 days cell culture, samples with an initial cell seeding density of 5 × 10^4^ cells/cm^2^ were incubated with viability/cytotoxicity assay for 30 min under the same conditions used for cell culture (37°C, humidified atmosphere with 5% CO_2_). Assay reagent-containing medium was replaced by fresh growth medium and imaging of samples was performed using an AxioImager microscope (Zeiss, Germany).

Cell alignment on 3D RGO/Alg scaffolds was analyzed, using ImageJ. While 0° was defined as cell horizontally orientated in an image, 90° was defined as cell vertically orientated in an image.

### Cell Proliferation Analysis

PrestoBlue assay (Thermo Fisher, Australia) was used for studying ADSC proliferation (initial seeding density: 4 × 10^4^ cells/cm^2^) in accordance to the manufacturer’s protocol. Fluorescence intensity of samples was measured in triplicate for each time point with a microplate reader (POLARstar Omega, Germany; excitation wavelength at 544 nm and emission wavelength at 590 nm).

### Expression of Alkaline Phosphatase Analysis

To quantitatively analyze alkaline phosphatase (ALP) upregulation in ADSCs on 3D and 2D substrates, ALP activity assay (Biovision, United States) was performed on days 3, 7, and 14 following cell seeding onto scaffolds. Osteogenic differentiation of ADSCs was induced in osteogenic differentiation medium (DIF medium) consisting of growth medium with 10 nM dexamethasone, 10 mM β-glycerophosphate and 50 μM L-ascorbic acid-2-phosphate (Kyllönen et al., 2013). Briefly, 3 × 10^5^ cells were seeded on each scaffold and cultured in DIF medium with medium refreshed every 2 days. On the day of assay, cells were lysed in 300 μl assay buffer for 30 min. Obtained lysis solution was centrifuged and resultant supernatant was reacted with 0.5 mM substrate solution for 30 min under light-proof conditions. The ALP activity of each sample was determined from the fluorescence measurement of formed fluorometric substrate using a microplate reader (POLARstar Omega, Germany; excitation wavelength at 360 nm and emission wavelength at 440 nm).

### Mineral Deposition Analysis

Firstly, ADSCs were seeded onto 3D and 2D substrates at a density of 4 × 10^4^ cells/cm^2^. Culture medium was changed every 2 days thereafter. After 3 weeks culture, 3.7% paraformaldehyde solution in phosphate-buffered saline (PBS) was used to fix samples with differentiated cells for 10 min. Fixed samples were then stained with 0.6% Alizarin Red S solution (pH 4.2) for 20 min at RT and washed with plentiful water. The stained cell mineral was eluted by acetic acid and methanol water solution (10%/20%) for 30 min, and eluted solution was transferred into 96-well plate for absorbance measurement in a microplate reader (POLARstar Omega, Germany) at 535 nm.

### Statistical Analysis

Unless specified, all data were expressed as mean ± SD. Prior to two-way ANOVA analysis (Bonferroni *post hoc* test, OriginPro 2015), homogeneity of variance tests (Levene’s test, OriginPro 2015) were performed to check the statistical assumptions were met. If homogeneity of variance was met (*P* > 0.05), statistical significance of two-way ANOVA analysis was set at *P* < 0.05. Otherwise (*P* < 0.05) statistical significance was set at *P* < 0.01 to increase the stringency.

## Results and Discussion

Initially, the components of the ink were characterized to determine properties likely to affect printability and/or subsequent physical properties of the 3D scaffolds to be produced.

### Rheological Properties of Alg/Gel

**FIGURE 2:**
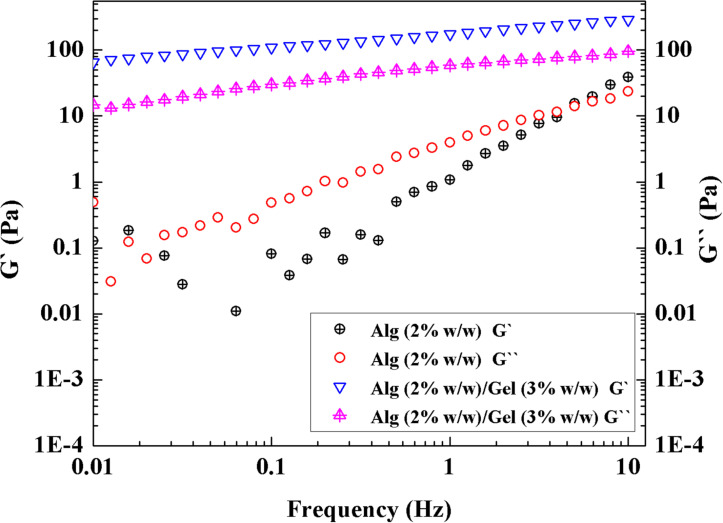
Rheology of Alg (2% w/w) solution and Alg (2% w/w)/Gel (3% w/w) ink with frequency sweep.

The rheological properties serve as an important indicator for extrudability and printability. Alg-based ink was prepared according to previously reported methods (Chung et al., 2013; Cheng et al., 2019). Rheological properties of Alg (2% w/w)/Gel (3% w/w) ink were tested by a dynamic frequency sweep and compared with that of Alg (2% w/w) solution without Gel component. As shown in Figure 2, Alg (2% w/w) solution showed higher loss modulus than storage modulus (G″ > G′) over most of the frequency range tested, indicative of a fluid rather than gel, and lacking appropriate viscoelastic properties for 3D printing. After Alg was blended with Gel, the Alg (2% w/w)/Gel (3% w/w) composite exhibited improved potential printability, as indicated by higher storage modulus than loss modulus (G′ > G″) across the frequencies tested (Chung et al., 2013).

### Characterization of GO and RGO

**FIGURE 3:**
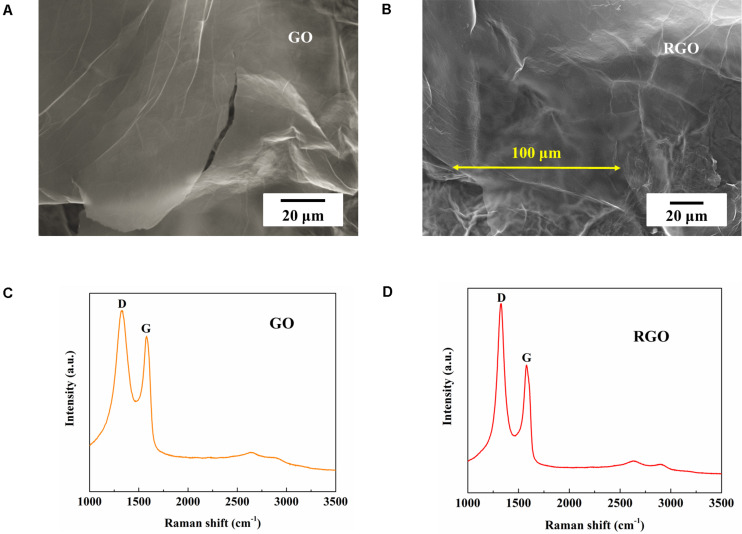
Characterization of synthesized GO and RGO. **(A,B)** SEM figures and **(C,D)** Raman spectra of synthesized GO and RGO respectively.

As-synthesized GO and RGO were characterized using SEM and Raman spectroscopy. Large layered structures of synthesized GO and RGO can be observed in the SEM images (Figures 3A,B) with a lateral size of more than 100 μm. As shown in Figures 3C,D, D and G band peaks at ∼1,330 and ∼1,580 cm^–1^ respective for Raman spectra are typically attributed to GO and RGO structures, and the intensity of D to G band ratio (*I*_D_/*I*_G_) are ∼1.18 and ∼1.52 for GO and RGO respectively, indicating defects introduced during the synthesis process and graphitization of GO after reduction (Gao and Tang, 2014).

### 3D Printing Alg/Gel Scaffolds

**FIGURE 4:**
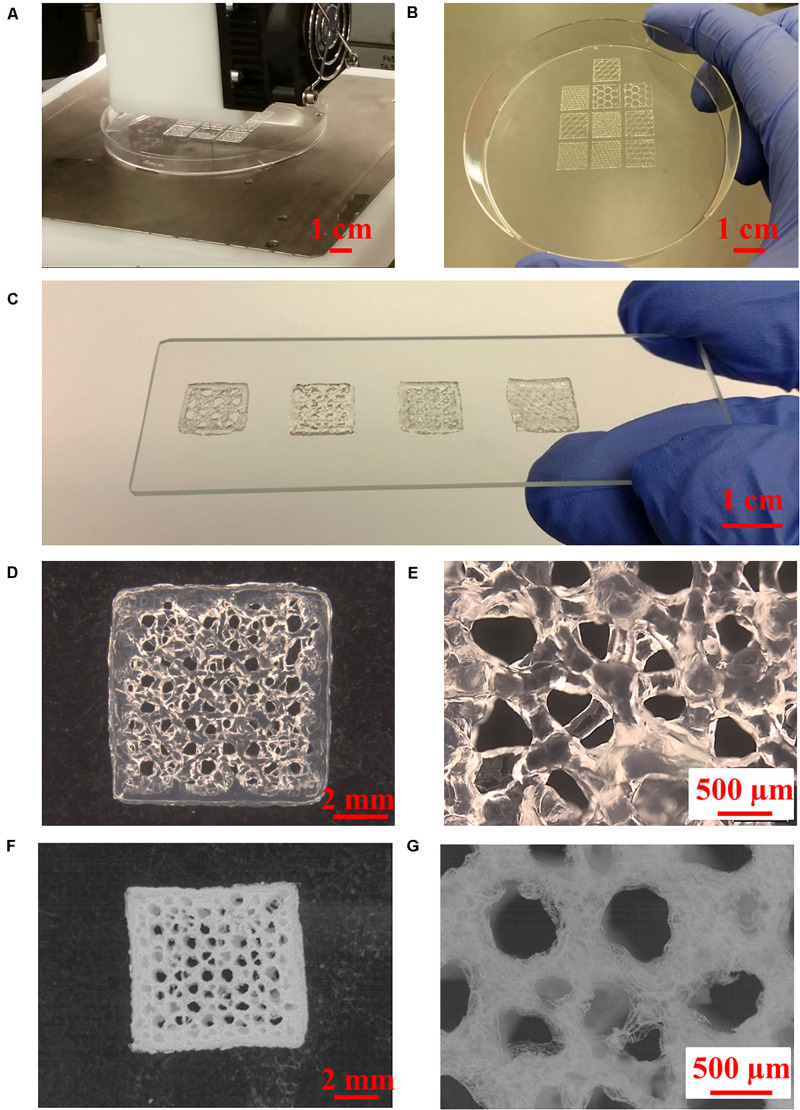
3D printing of Alg/Gel composite scaffolds. **(A)** High-throughput 3D printing of multiple Alg/Gel composite scaffolds into a petri dish, **(B)** 3D printed Alg/Gel composite scaffolds with different inner structures. **(C)** Multi-angle 3D printed Alg/Gel composite scaffolds with different interstrand distances (from left to right: 2, 1.5, 1, and 0.5 mm). **(D,E)** Multi-angle 3D printed Alg/Gel composite scaffold with interstrand distance of 1.5 mm in hydrated state at low and high magnification. **(F,G)** Multi-angle 3D printed Alg/Gel composite scaffold with interstrand distance of 1.5 mm in freeze-dried state at low and high magnification.

3D scaffolds with controlled inner structural patterns and interstrand distances (0.5–2 mm) can be printed simultaneously, as shown in Figures 4A–C. Cell support and tissue regeneration can be facilitated with manipulation of the geometry and porosity of a scaffold to allow sufficient supply of nutrition and oxygen (Woo Jung et al., 2013). The 3D printed scaffolds showed well-defined architecture both in hydrated and freeze-dried states (Figures 4D–G).

**FIGURE 5:**
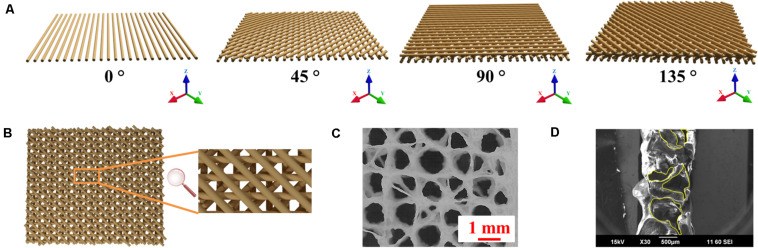
3D printing schema. **(A)** Schematic of fabrication of multi-angle 3D printed Alg/Gel scaffold. **(B)** Top view of four-layer model of 3D printed Alg/Gel scaffold. **(C)** Photomicrograph of top view of freeze-dried multi-angle 3D Alg scaffold. **(D)** SEM image of cross-sectional view of 3D Alg scaffold with horizontal pores highlighted by yellow contours.

The orientation of the extruded strands is important for 3D printing Alg-based scaffolds as this provides the basis for structural stability (He et al., 2016). As shown in Supplementary Figures S1A–D, 3D printed Alg scaffolds with traditional 90° angle (cruciform) collapse during printing, reducing porosity with limited transport of nutrients and waste. The use of multi-angle 3D printing has been employed here and involves rearranging the print orientation of subsequent layers such that printed strands have a 45° angle clockwise to the preceding layer and every fourth layer structure is a repeat of the initial pattern (Figure 5A). Using this method, the contact area between strands in adjacent layers is increased compared with the traditional 90° 3D printed scaffold by about 41.4%, resulting in enhanced structural stability of the scaffold (Figure 5B). The revised structure contains interconnected pores with a wide pore size distribution (from ∼100 to ∼1,000 μm) due to multi-angle printing strategy. It‘s reported that small porosity permits effective cell signaling and attachment, but ineffective for oxygen and nutrient supply (Yang et al., 2002; Oh et al., 2007). Large porosity is useful for the opposite properties, so the fabricated scaffold with gradient pore sizes should be beneficial for tissue regeneration (Hutmacher, 2000). Porosity in the x, y, and z phases (Figures 4E, 5C,D) is important for applications in tissue engineering, since this facilitates access to nutrients and waste product removal, while providing channels for vascularization and waste removal (Bose et al., 2012).

### Graphene Coating

**FIGURE 6:**
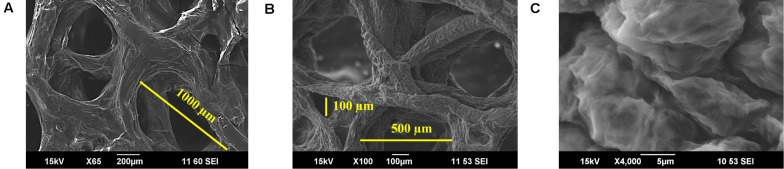
Characterization of 3D RGO/Alg scaffolds. **(A–C)** SEM images of 3D RGO/Alg at different magnifications.

Using the procedures detailed in the experimental section, uniform and adhered coating of GO was obtained. The coating remained uniform and adherent after the chemical reduction process. SEM images at different magnifications confirm uniform coating of RGO on the scaffolds and the integrity of interconnected pores, as shown in Figures 6A–C. Pores of scaffolds varied from 100 μm to about 1,000 μm diameter, as illustrated in Figures 6A,B. After coating with RGO, elastic modulus of 3D RGO/Alg scaffolds increased 3.8-fold compared to Alg-only scaffolds (Supplementary Figure S2), which is consistent with the previously reported reinforcing property of graphene (Güler and Bağc1, 2020). The RGO/Alg scaffold was electrically conductive as further evidence of successful RGO coating, with a sheet resistance of 1.5 kΩ/sq (±0.14). *I*_D_/*I*_G_ ratio of 3D RGO/Alg scaffold was measured to be 1.54 (Supplementary Figure S3), indicative of the quality of RGO comparable to the RGO synthesized directly.

### ADSC Culture and Differentiation

**FIGURE 7:**
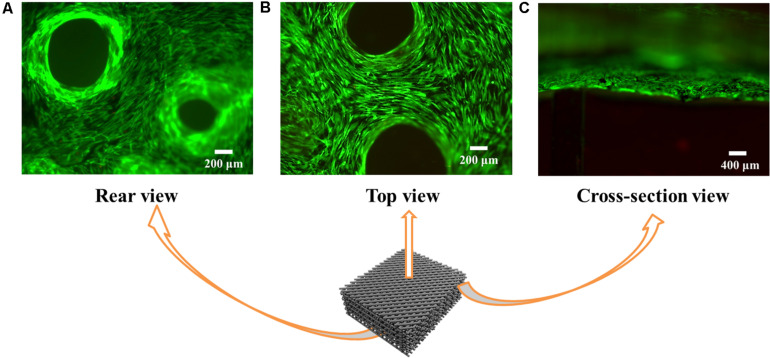
Cell viability and adhesion on the 3D RGO/Alg scaffolds. Fluorescence microscope images of live/dead ADSC staining on 3D RGO/Alg scaffolds from **(A)** rear view, **(B)** top view and **(C)** cross-sectional view following 7 days culture.

Cells do not adhere well to Alg due to absence of adhesion molecules (Rowley et al., 1999). Here we find that coating graphene on the Alg scaffold promotes cell adhesion. As shown in Figures 7A–C, the majority of cells cultured on 3D RGO/Alg scaffolds were viable after 7 days. Notably, even though cells were initially seeded on the top of the scaffolds, scaffolds were completely covered by cells after 7 days. This indicated high cell affinity for the scaffolds and vigorous cell migration. As indicated by Supplementary Figures S4A,B, cells attached to the scaffold surface efficiently by filopodia.

**FIGURE 8:**
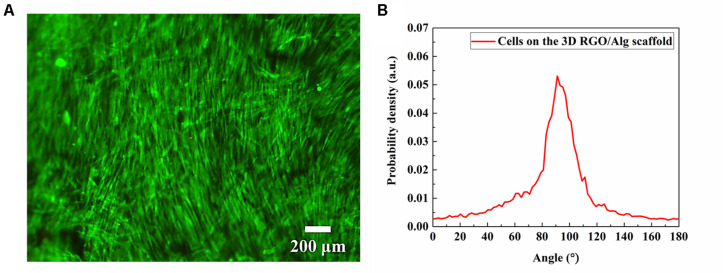
Cell alignment analysis. **(A)** Fluorescence microscope images of live/dead ADSC staining showing cell alignment on a 3D RGO/Alg scaffold influenced by localized geometric factors following 7 day culture, and **(B)** quantitative analysis of cell orientation.

As shown in Figures 8A,B, cells showed alignment on 3D RGO/Alg scaffolds, owing to the wrinkled features of graphene coating (Figures 6A,B and Supplementary Figures S4A,B). Alignment of cells can provide mechanotransductive signals, such as integrin altered intracellular forces, clustering, and cytoskeletal organization (Newman et al., 2016). Spatial alignment of cells can result in traction forces to direct expression of fusion proteins and fusion behaviors (Choi et al., 2012). The property may induce osteogenic differentiation and be utilized to manipulate cell behavior via adjusting subtle geometric features of 3D scaffolds.

**FIGURE 9:**
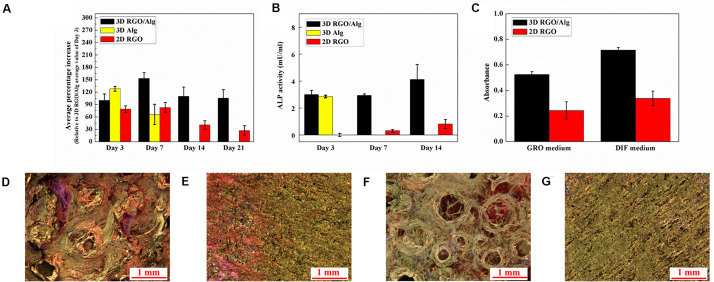
Proliferation and osteogenic differentiation of ADSCs on 3D RGO/Alg scaffold. **(A)** ADSC proliferation on different 3D and 2D substrates indicated by average percentage increase of fluorescence intensity (relative to Day 3 average value of 3D RGO/Alg samples) over time. Mean ± SD, *n* = 3 [two-way ANOVA, *F*(6,60) = 31.56, *P* < 0.0001; Bonferroni *post hoc*, *P* < 0.01 (3D RGO/Alg Day 7 vs all comparisons except for 3D Alg Day 3; 3D Alg Day 3 vs 3D Alg Day 7; 2D RGO Day 3 and 7 vs 2D RGO Day 14 and 21; 3D RGO/Alg vs 2D RGO on Day 14 and 21; 3D Alg Day 3 vs 2D RGO Day 3, 14 and 21)]. **(B)** Alkaline phosphatase (ALP) expression of differentiating ADSCs on 3D constructs and 2D substrates at different time points. Mean ± SD, *n* = 3 [two-way ANOVA, *F*(4,45) = 49.21, *P* < 0.0001; Bonferroni *post hoc*, *P* < 0.01 (3D RGO/Alg Day 14 vs all the comparisons; 2D RGO Day 3, 7, and 14 vs all the 3D comparisons)]. **(C)** Quantification of mineral deposition, with results shown as mean ± SD, *n* = 3 [two-way ANOVA, *F*(1,20) = 6.27, *P* = 0.021; Bonferroni *post hoc*, *P* < 0.01 (3D RGO/Alg DIF medium vs all the comparisons; 3D RGO/Alg GRO medium vs 2D RGO GRO medium)]. Mineral deposition of ADSCs cultured in differentiation medium on **(D)** 3D RGO/Alg scaffold and **(E)** 2D RGO substrate paper, or in growth medium on **(F)** 3D RGO/Alg scaffold and **(G)** 2D RGO substrate for 3 weeks. Samples were stained with Alizarin Red S.

ADSC proliferation on both 3D and 2D substrates was assessed using PrestoBlue assay. Cell proliferation on 2D RGO substrate was performed to compare 2D planar verses 3Dscaffold-based cell support. During testing, 3D Alg scaffolds dissolved in culture medium following Day 7 of culture. As such, no data was available for 3D Alg samples thereafter. Notwithstanding, as shown in Figure 9A, greater numbers of cells grew on 3D scaffolds compared to 2D substrates from Day 3 to Day 21, with cell numbers on Days 7, 14, and 21 on 3D RGO/Alg scaffolds being significantly higher than on 2D substrates (*P* < 0.05). These findings were consistent with our previous published data showing increased cell proliferation on 3D construct (Li et al., 2017). The number of cells that grew on 3D RGO/Alg scaffolds and 2D RGO substrates peaked on Day 7, and Day 3 for 3D Alg scaffolds. Peak cell-growth for 3D RGO/Alg scaffolds was about 20 and 85% higher than that of 3D Alg scaffolds and 2D RGO substrates, respectively. Statistical analysis indicated that both cell supporting structure [*F*(2,60) = 161.75, *P* < 0.0001] and day [*F*(3,60) = 86.44, *P* < 0.0001] significantly affect cell proliferation, as well as the interaction of cell supporting structure and day [Overall two-way ANOVA, *F*(6,60) = 31.56, *P* < 0.0001]. Specifically, Bonferroni *post hoc* analysis indicated that proliferation of ADSCs on 3D RGO/Alg scaffolds was significantly different from that on the other cell supports (*P* < 0.01). Therefore, the 3D RGO/Alg scaffolds are mechanically more robust in a cell culture environment and provide better cell support than 3D Alg-only scaffolds and 2D RGO substrates.

Osteogenic induction of ADSCs was affected by 3D architecture and graphene substrate as determined by ALP (an important osteogenic differentiation marker) expression at different time points (Figure 9B). Statistical analysis revealed a significant effect of cell support [*F*(2,45) = 267.86, *P* < 0.0001] and day [*F*(2,45) = 21.00, *P* < 0.0001], as well as the interaction between cell support and day [Overall two-way ANOVA, *F*(4,45) = 49.21, *P* < 0.0001]. Particularly, Bonferroni-*post hoc* analysis revealed ALP expression of cells on 3D RGO/Alg scaffolds at Day 14 was significantly higher compared to all other comparisons, while ALP expression of cells on all the 3D scaffolds was significantly higher compared to 2D RGO substrate. The peak value for ALP expression for 3D RGO/Alg scaffolds was 5 times that of 2D RGO substrates, supporting osteogenic differentiation of stem cells. Due to the de-crosslinking effect of differentiation medium, 3D Alg-only scaffolds were dissolved in the culture medium after Day 3, resulting in the inability to generate data thereafter.

Mineral deposition by cells on different structures during osteogenic differentiation was investigated by Alizarin Red S staining, as shown in Figures 9C–G. Greater mineral deposition was observed for 3D scaffolds compared to 2D substrates. Statistical analysis revealed there was a significant effect of cell support [*F*(1,20) = 301.21, *P* < 0.0001] and culture medium [*F*(1,20) = 56.45, *P* < 0.0001], but not the interaction between cell support and culture medium [Overall two-way ANOVA, *F*(1,20) = 6.27, *P* = 0.021]. Particularly, Bonferroni-*post hoc* analysis and quantification of stained mineral deposition (Figure 9C) revealed that mineral deposition on the 3D RGO/Alg scaffolds in differentiation medium was significantly higher compared to all the comparisons. This finding is consistent with previous reports on osteogenic induction of stem cells by 3D graphene scaffolds (Crowder et al., 2013; Palmieri et al., 2020). It is worth noting that L-ascorbic acid-2-phosphate in differentiation medium can de-crosslink 3D Alg scaffolds (totally dissolving after Day 3), but RGO coated 3D Alg scaffolds remain intact for over 3 weeks due to the protective RGO coating. The mechanism underlying osteogenesis of stem cells likely relates to activation of the mechanosensitive integrin/FAK axis by graphene (Xie et al., 2019). Subtle geometric features of the 3D RGO/Alg scaffold may also influence the spatial alignment of cells, contributing to ADSC differentiation (Kilian et al., 2010).

In summary, ADSCs favored culture and differentiation on 3D constructs. After RGO coating, 3D Alg scaffolds exhibited improved stability in the cell culture environment. Especially with the flexibility and customization of 3D printing, our developed 3D graphene constructs possess great potential for personalized tissue regeneration in clinic.

## Conclusion

We have synthesized 3D RGO/Alg scaffolds by combining advanced 3D printing with material-blending and traditional graphene coating method. Multi-angle 3D printing was utilized to fabricate 3D Alg-based scaffolds without collapse and fusion, enabling scaffolds with various pore shapes and sizes to be fabricated together with simple GO deposition for coating. Coating of RGO on 3D Alg scaffolds further increased mechanical strength and cytocompatibility, with successful coating further evidenced by electrical conductivity. Our as-fabricated 3D RGO/Alg scaffolds showed enhanced ADSC support and osteogenic differentiation compared to 2D RGO substrates, as proof-of-concept for bone-engineering and potential application for *in vivo* bone regeneration. Moreover, our scaffold may be adapted to support a range of cell-types and engineer a variety of tissues for research and translation.

## Data Availability Statement

The raw data supporting the conclusions of this article will be made available by the authors, without undue reservation.

## Author Contributions

JL, XL, GW, and JC conceived the study. JL executed the experiments. JL, JC, and GW wrote the manuscript. JC and GW were co-senior corresponding authors. All authors contributed to the article and approved the submitted version.

## Conflict of Interest

The authors declare that the research was conducted in the absence of any commercial or financial relationships that could be construed as a potential conflict of interest.
