# Supplementary material for: 3D Printing of Cytocompatible Graphene/Alginate Scaffolds for Mimetic Tissue Constructs
Source: Front Bioeng Biotechnol. 2020 Jul 17;8:824. doi: 10.3389/fbioe.2020.00824 (PMC7379132; doi:10.3389/fbioe.2020.00824)
Supplement: Supplementary file 1 [file Data_Sheet_1.docx]

**3D Printing of Cytocompatible Graphene/Alginate Scaffolds for Mimetic Tissue Constructs**

Jianfeng Li^1^, Xiao Liu^1^, Jeremy M. Crook^1,2,3^*, and Gordon G. Wallace^1^*

^1^ARC Centre of Excellence for Electromaterials Science,

Intelligent Polymer Research Institute, AIIM Facility,

University of Wollongong, NSW 2500, Australia

^2^Illawarra Health and Medical Research Institute, University of Wollongong, Wollongong, New South Wales 2522, Australia

^3^Department of Surgery, St Vincent’s Hospital, The University of Melbourne, Fitzroy, Victoria 3065, Australia

*Corresponding authors E-mail addresses: jcrook@uow.edu.au; gwallace@uow.edu.au

**Supporting Information**




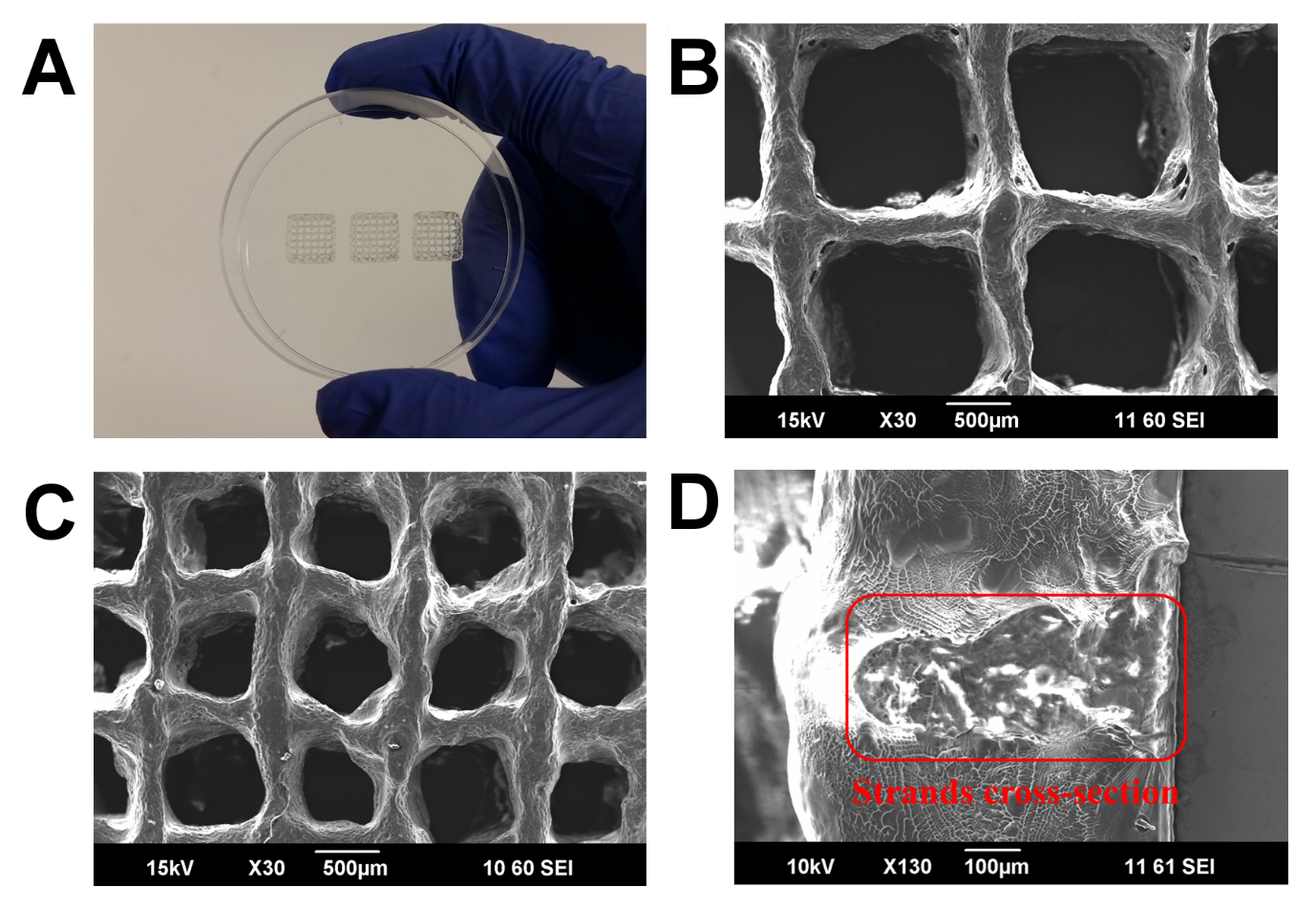
**FIGURE S1 |** **(A)** Figure of 3D printed Alg/Gel scaffold with cruciform strand arrangement. **(B, C)** SEM images of 3D printed Alg-only scaffold with cruciform strand arrangement and different interstrand distance. **(D)** SEM images of cross section structure of stacked strands in 3D Alg scaffold.

**FIGURE S2 |** Stress-strain curves for 3D Alg and 3D RGO/Alg scaffolds.





**FIGURE S3 |** Raman spectrum of synthesized 3D RGO/Alg scaffold.


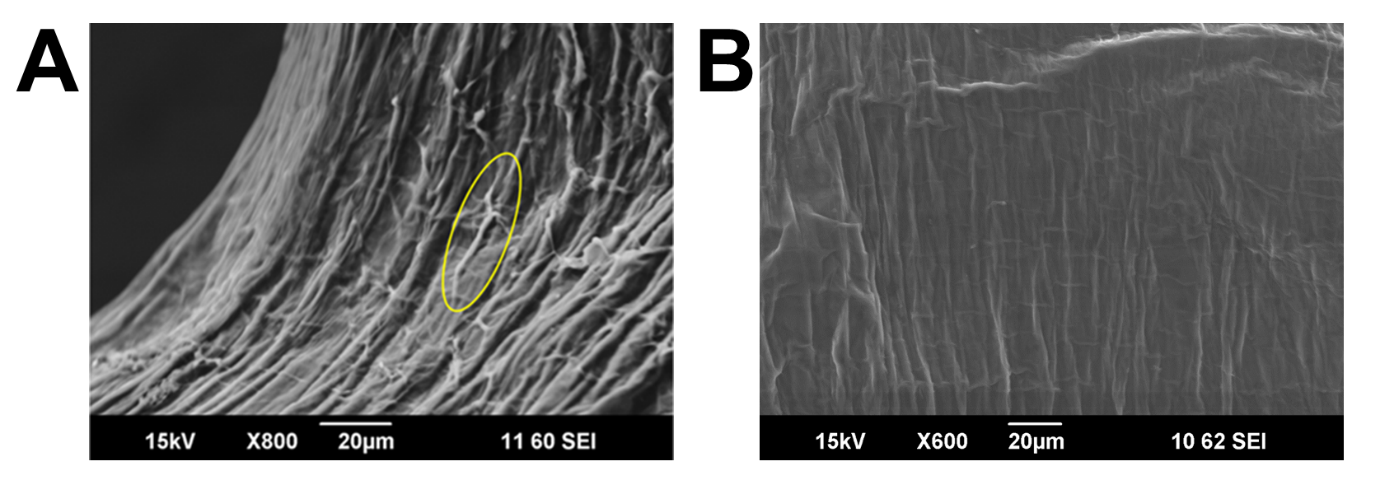


**FIGURE S4 |** (A) SEM of ADSCs on a 3D RGO/Alg scaffold after one day culture. A representative cell is highlighted (yellow circle). (B) SEM of a 3D RGO/Alg scaffold without cells.
